# 1-(2-Chloro­phenyl)-6-fluoro-2-methyl-1*H*-indole-3-carbonitrile

**DOI:** 10.1107/S1600536811011214

**Published:** 2011-04-07

**Authors:** Kun Yang, Pei-Fan Li, Yan Liu, Zhi-Zhong Fang

**Affiliations:** a Teaching & Research Center, Tianjin Medical University, Tianjin 300070, People’s Republic of China; b Pharmacy Department, Tianjin Medical College, Tianjin 300222, People’s Republic of China

## Abstract

In the title compound, C_16_H_10_ClFN_2_, the dihedral angle between the indole ring system and the benzyl ring is 80.91 (5)°. The crystal packing features C—H⋯Cl, C—H⋯F and C—H⋯π inter­actions.

## Related literature

For the synthesis of the title compound, see: Du *et al.* (2006 ▶). For its precursor, see: Jin *et al.* (2009 ▶). For related structures, see: Li & Huang (2009 ▶); Li *et al.* (2009 ▶, 2010*a* ▶,*b* ▶).
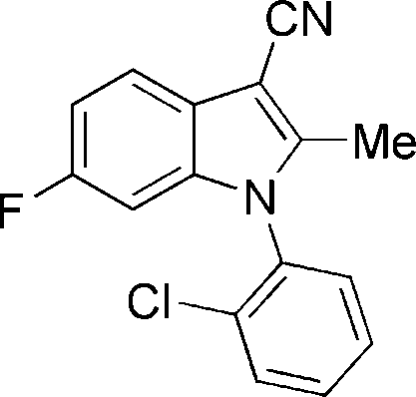


## Experimental

### 

#### Crystal data

C_16_H_10_ClFN_2_
*M* *_r_* = 284.71
Orthorhombic, 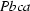

*a* = 7.4581 (9) Å
*b* = 16.8480 (15) Å
*c* = 21.356 (2) Å
*V* = 2683.5 (5) Å^3^
*Z* = 8
Mo *K*α radiation
μ = 0.29 mm^−1^
*T* = 113 K
0.26 × 0.22 × 0.20 mm

#### Data collection

Rigaku Saturn724 CCD diffractometer
Absorption correction: multi-scan (*CrystalClear*; Rigaku, 2009 ▶) *T*_min_ = 0.929, *T*_max_ = 0.945
28426 measured reflections
3893 independent reflections
3219 reflections with *I* > 2σ(*I*)
*R*_int_ = 0.034

#### Refinement

*R*[*F* ^2^ > 2σ(*F* ^2^)] = 0.041
*wR*(*F* ^2^) = 0.113
*S* = 1.11
3893 reflections
182 parameters
H-atom parameters constrained
Δρ_max_ = 0.39 e Å^−3^
Δρ_min_ = −0.20 e Å^−3^

### 

Data collection: *CrystalClear* (Rigaku, 2009 ▶); cell refinement: *CrystalClear*; data reduction: *CrystalClear*; program(s) used to solve structure: *SHELXS97* (Sheldrick, 2008 ▶); program(s) used to refine structure: *SHELXL97* (Sheldrick, 2008 ▶); molecular graphics: *CrystalStructure* (Rigaku, 2009 ▶); software used to prepare material for publication: *CrystalStructure*.

## Supplementary Material

Crystal structure: contains datablocks global, I. DOI: 10.1107/S1600536811011214/hb5823sup1.cif

Structure factors: contains datablocks I. DOI: 10.1107/S1600536811011214/hb5823Isup2.hkl

Additional supplementary materials:  crystallographic information; 3D view; checkCIF report

## Figures and Tables

**Table 1:** Hydrogen-bond geometry (Å, °) *Cg*1 is the centroid of the C3–C8 benzene ring.

*D*—H⋯*A*	*D*—H	H⋯*A*	*D*⋯*A*	*D*—H⋯*A*
C7—H7⋯F1^i^	0.95	2.54	3.1638 (16)	123
C7—H7⋯Cl1^ii^	0.95	2.73	3.5296 (14)	142
C15—H15⋯*Cg*1^iii^	0.95	2.92	3.7246 (14)	143

Symmetry codes: (i) 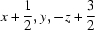; (ii) 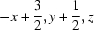; (iii) 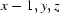.

**Fig. 1.**
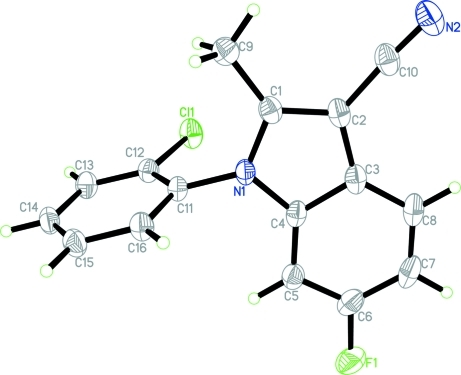
The molecular structure of (I) with 50% probability displacement ellipsoids.

## supplementary crystallographic information

### Comment

Indoles are an important compound possessing pharmaceutical properties. Extensive investigation on the crystal structures of indoles helps disclose their structure-activity relationship. For continuing our reseach, herein, we reported the crystal structure of the title indole derivative.

In the molecular structure, (I) (Fig. 1), the indole ring system is almost planar with a dihedral angle of 0.85 (6)° between its pyrrole ring and fused benzene ring. The indole ring forms an angle of 80.91 (5)° with the chlorobenzene ring.

### Experimental

The title compound was prepared according to the method of the literature (Du, *et al.*, 2006). Colourless prisms were grown from a mixture of ethyl actate and petroleum ether.

### Refinement

All H atoms were positioned geometrically (C—H = 0.95 and 0.98 Å)and refined as riding with *U*_iso_(H) = 1.2*U*_eq_(CH) or 1.5*U*_eq_(CH_3_).

### Crystal data

**Table:**

C_16_H_10_ClFN_2_	*F*(000) = 1168
*M**_r_* = 284.71	*D*_x_ = 1.409 Mg m^−^^3^
Orthorhombic, *P**b**c**a*	Mo *K*α radiation, λ = 0.71075 Å
Hall symbol: -P 2ac 2ab	Cell parameters from 10281 reflections
*a* = 7.4581 (9) Å	θ = 1.5–31.4°
*b* = 16.8480 (15) Å	µ = 0.29 mm^−^^1^
*c* = 21.356 (2) Å	*T* = 113 K
*V* = 2683.5 (5) Å^3^	Prism, colorless
*Z* = 8	0.26 × 0.22 × 0.20 mm

### Data collection

**Table:**

Rigaku Saturn724 CCD diffractometer	3893 independent reflections
Radiation source: rotating anode	3219 reflections with *I* > 2σ(*I*)
multilayer	*R*_int_ = 0.034
Detector resolution: 14.222 pixels mm^-1^	θ_max_ = 30.0°, θ_min_ = 1.9°
ω scans	*h* = −10→9
Absorption correction: multi-scan (*CrystalClear*; Rigaku, 2009)	*k* = −23→23
*T*_min_ = 0.929, *T*_max_ = 0.945	*l* = −30→30
28426 measured reflections	

### Refinement

**Table:**

Refinement on *F*^2^	Primary atom site location: structure-invariant direct methods
Least-squares matrix: full	Secondary atom site location: difference Fourier map
*R*[*F*^2^ > 2σ(*F*^2^)] = 0.041	Hydrogen site location: inferred from neighbouring sites
*wR*(*F*^2^) = 0.113	H-atom parameters constrained
*S* = 1.11	*w* = 1/[σ^2^(*F*_o_^2^) + (0.0644*P*)^2^ + 0.1004*P*] where *P* = (*F*_o_^2^ + 2*F*_c_^2^)/3
3893 reflections	(Δ/σ)_max_ = 0.001
182 parameters	Δρ_max_ = 0.39 e Å^−^^3^
0 restraints	Δρ_min_ = −0.20 e Å^−^^3^

### Special details

**Table:**

Geometry. All e.s.d.'s (except the e.s.d. in the dihedral angle between two l.s. planes) are estimated using the full covariance matrix. The cell e.s.d.'s are taken into account individually in the estimation of e.s.d.'s in distances, angles and torsion angles; correlations between e.s.d.'s in cell parameters are only used when they are defined by crystal symmetry. An approximate (isotropic) treatment of cell e.s.d.'s is used for estimating e.s.d.'s involving l.s. planes.
Refinement. Refinement of *F*^2^ against ALL reflections. The weighted *R*-factor *wR* and goodness of fit *S* are based on *F*^2^, conventional *R*-factors *R* are based on *F*, with *F* set to zero for negative *F*^2^. The threshold expression of *F*^2^ > σ(*F*^2^) is used only for calculating *R*-factors(gt) *etc*. and is not relevant to the choice of reflections for refinement. *R*-factors based on *F*^2^ are statistically about twice as large as those based on *F*, and *R*- factors based on ALL data will be even larger.

### Fractional atomic coordinates and isotropic or equivalent isotropic displacement parameters (Å^2^)

**Table:**

	*x*	*y*	*z*	*U*_iso_*/*U*_eq_	
Cl1	0.50520 (4)	0.220294 (19)	0.641958 (16)	0.03226 (11)	
F1	0.61121 (11)	0.54670 (5)	0.76153 (4)	0.0480 (2)	
N1	0.39260 (12)	0.37712 (5)	0.59488 (4)	0.0236 (2)	
N2	0.79477 (16)	0.43384 (7)	0.42798 (6)	0.0420 (3)	
C1	0.45553 (17)	0.36559 (7)	0.53489 (5)	0.0258 (2)	
C2	0.60108 (16)	0.41469 (7)	0.52619 (6)	0.0271 (3)	
C3	0.63160 (15)	0.45816 (7)	0.58328 (6)	0.0265 (3)	
C4	0.49938 (14)	0.43272 (7)	0.62558 (6)	0.0237 (2)	
C5	0.48747 (16)	0.46061 (7)	0.68659 (6)	0.0280 (3)	
H5	0.3986	0.4427	0.7151	0.034*	
C6	0.61408 (17)	0.51607 (7)	0.70243 (6)	0.0337 (3)	
C7	0.74735 (17)	0.54359 (8)	0.66235 (7)	0.0361 (3)	
H7	0.8315	0.5819	0.6765	0.043*	
C8	0.75667 (16)	0.51503 (7)	0.60210 (7)	0.0318 (3)	
H8	0.8460	0.5336	0.5740	0.038*	
C9	0.36953 (19)	0.30813 (8)	0.49124 (6)	0.0333 (3)	
H9A	0.3812	0.2543	0.5081	0.040*	
H9B	0.4286	0.3109	0.4503	0.040*	
H9C	0.2423	0.3213	0.4866	0.040*	
C10	0.70533 (17)	0.42350 (7)	0.47083 (6)	0.0317 (3)	
C11	0.25337 (16)	0.33290 (7)	0.62467 (5)	0.0223 (2)	
C12	0.28935 (15)	0.25756 (7)	0.64802 (5)	0.0230 (2)	
C13	0.15588 (16)	0.21330 (7)	0.67690 (5)	0.0271 (3)	
H13	0.1800	0.1612	0.6918	0.032*	
C14	−0.01309 (16)	0.24642 (8)	0.68363 (6)	0.0277 (3)	
H14	−0.1052	0.2169	0.7037	0.033*	
C15	−0.04958 (18)	0.32238 (7)	0.66141 (6)	0.0295 (3)	
H15	−0.1658	0.3446	0.6664	0.035*	
C16	0.08411 (16)	0.36557 (7)	0.63194 (6)	0.0276 (3)	
H16	0.0598	0.4175	0.6167	0.033*	

### Atomic displacement parameters (Å^2^)

**Table:**

	*U*^11^	*U*^22^	*U*^33^	*U*^12^	*U*^13^	*U*^23^
Cl1	0.02051 (19)	0.02941 (18)	0.0468 (2)	0.00492 (11)	0.00001 (12)	0.00824 (12)
F1	0.0450 (5)	0.0454 (5)	0.0535 (5)	−0.0100 (4)	−0.0018 (4)	−0.0201 (4)
N1	0.0198 (5)	0.0213 (4)	0.0298 (5)	−0.0031 (4)	0.0030 (4)	0.0022 (4)
N2	0.0363 (7)	0.0436 (7)	0.0461 (6)	0.0006 (5)	0.0128 (5)	0.0089 (5)
C1	0.0240 (6)	0.0243 (5)	0.0290 (5)	0.0016 (5)	0.0019 (5)	0.0054 (4)
C2	0.0218 (6)	0.0250 (5)	0.0344 (6)	0.0014 (4)	0.0040 (5)	0.0079 (5)
C3	0.0199 (6)	0.0210 (5)	0.0385 (6)	0.0020 (4)	0.0007 (5)	0.0081 (4)
C4	0.0178 (6)	0.0181 (5)	0.0353 (6)	0.0001 (4)	−0.0013 (4)	0.0036 (4)
C5	0.0234 (6)	0.0230 (6)	0.0376 (6)	−0.0001 (4)	0.0013 (5)	0.0006 (5)
C6	0.0311 (7)	0.0253 (6)	0.0446 (7)	0.0007 (5)	−0.0046 (6)	−0.0057 (5)
C7	0.0267 (7)	0.0220 (6)	0.0597 (8)	−0.0050 (5)	−0.0061 (6)	0.0016 (6)
C8	0.0199 (6)	0.0225 (6)	0.0528 (7)	−0.0007 (4)	0.0011 (6)	0.0100 (5)
C9	0.0349 (7)	0.0345 (7)	0.0304 (6)	−0.0037 (6)	0.0011 (5)	0.0007 (5)
C10	0.0259 (7)	0.0291 (6)	0.0402 (6)	0.0026 (5)	0.0047 (5)	0.0088 (5)
C11	0.0194 (6)	0.0216 (5)	0.0259 (5)	−0.0029 (4)	0.0004 (4)	0.0022 (4)
C12	0.0188 (6)	0.0235 (5)	0.0267 (5)	0.0008 (4)	−0.0021 (4)	0.0014 (4)
C13	0.0263 (6)	0.0255 (5)	0.0295 (5)	−0.0022 (5)	−0.0015 (5)	0.0070 (4)
C14	0.0230 (6)	0.0324 (6)	0.0275 (5)	−0.0069 (5)	0.0020 (5)	0.0031 (5)
C15	0.0199 (6)	0.0318 (6)	0.0369 (6)	0.0006 (5)	0.0032 (5)	−0.0002 (5)
C16	0.0229 (6)	0.0241 (6)	0.0358 (6)	0.0015 (5)	0.0009 (5)	0.0041 (5)

### Geometric parameters (Å, °)

**Table:**

Cl1—C12	1.7328 (12)	C7—C8	1.375 (2)
F1—C6	1.3636 (15)	C7—H7	0.9500
N1—C1	1.3780 (15)	C8—H8	0.9500
N1—C4	1.3934 (14)	C9—H9A	0.9800
N1—C11	1.4276 (14)	C9—H9B	0.9800
N2—C10	1.1457 (16)	C9—H9C	0.9800
C1—C2	1.3775 (16)	C11—C16	1.3857 (16)
C1—C9	1.4892 (17)	C11—C12	1.3900 (15)
C2—C10	1.4227 (17)	C12—C13	1.3883 (16)
C2—C3	1.4404 (18)	C13—C14	1.3857 (17)
C3—C8	1.3963 (17)	C13—H13	0.9500
C3—C4	1.4045 (16)	C14—C15	1.3918 (18)
C4—C5	1.3879 (18)	C14—H14	0.9500
C5—C6	1.3708 (17)	C15—C16	1.3855 (17)
C5—H5	0.9500	C15—H15	0.9500
C6—C7	1.3914 (19)	C16—H16	0.9500
			
C1—N1—C4	109.73 (9)	C3—C8—H8	120.8
C1—N1—C11	126.05 (10)	C1—C9—H9A	109.5
C4—N1—C11	123.83 (9)	C1—C9—H9B	109.5
C2—C1—N1	108.01 (10)	H9A—C9—H9B	109.5
C2—C1—C9	130.19 (11)	C1—C9—H9C	109.5
N1—C1—C9	121.80 (11)	H9A—C9—H9C	109.5
C1—C2—C10	127.27 (12)	H9B—C9—H9C	109.5
C1—C2—C3	108.40 (10)	N2—C10—C2	176.12 (15)
C10—C2—C3	124.33 (11)	C16—C11—C12	119.89 (10)
C8—C3—C4	119.56 (12)	C16—C11—N1	120.35 (10)
C8—C3—C2	134.28 (11)	C12—C11—N1	119.73 (10)
C4—C3—C2	106.16 (10)	C13—C12—C11	120.76 (11)
C5—C4—N1	129.26 (11)	C13—C12—Cl1	120.31 (9)
C5—C4—C3	123.06 (11)	C11—C12—Cl1	118.91 (9)
N1—C4—C3	107.68 (11)	C14—C13—C12	118.81 (11)
C6—C5—C4	114.73 (11)	C14—C13—H13	120.6
C6—C5—H5	122.6	C12—C13—H13	120.6
C4—C5—H5	122.6	C13—C14—C15	120.85 (11)
F1—C6—C5	118.39 (12)	C13—C14—H14	119.6
F1—C6—C7	117.04 (11)	C15—C14—H14	119.6
C5—C6—C7	124.57 (13)	C16—C15—C14	119.80 (12)
C8—C7—C6	119.68 (12)	C16—C15—H15	120.1
C8—C7—H7	120.2	C14—C15—H15	120.1
C6—C7—H7	120.2	C15—C16—C11	119.86 (11)
C7—C8—C3	118.39 (12)	C15—C16—H16	120.1
C7—C8—H8	120.8	C11—C16—H16	120.1
			
C4—N1—C1—C2	−1.08 (13)	C4—C5—C6—C7	−0.48 (18)
C11—N1—C1—C2	−174.11 (10)	F1—C6—C7—C8	−179.81 (11)
C4—N1—C1—C9	178.91 (11)	C5—C6—C7—C8	0.4 (2)
C11—N1—C1—C9	5.89 (17)	C6—C7—C8—C3	−0.63 (18)
N1—C1—C2—C10	−178.61 (11)	C4—C3—C8—C7	0.91 (17)
C9—C1—C2—C10	1.4 (2)	C2—C3—C8—C7	−178.87 (13)
N1—C1—C2—C3	0.59 (13)	C1—C2—C10—N2	177 (100)
C9—C1—C2—C3	−179.40 (12)	C3—C2—C10—N2	−2(2)
C1—C2—C3—C8	179.90 (13)	C1—N1—C11—C16	−104.36 (13)
C10—C2—C3—C8	−0.9 (2)	C4—N1—C11—C16	83.56 (14)
C1—C2—C3—C4	0.10 (13)	C1—N1—C11—C12	77.42 (15)
C10—C2—C3—C4	179.33 (11)	C4—N1—C11—C12	−94.66 (13)
C1—N1—C4—C5	−178.37 (12)	C16—C11—C12—C13	2.09 (17)
C11—N1—C4—C5	−5.16 (18)	N1—C11—C12—C13	−179.69 (10)
C1—N1—C4—C3	1.15 (13)	C16—C11—C12—Cl1	−176.62 (9)
C11—N1—C4—C3	174.36 (10)	N1—C11—C12—Cl1	1.61 (15)
C8—C3—C4—C5	−1.03 (17)	C11—C12—C13—C14	−1.76 (17)
C2—C3—C4—C5	178.81 (11)	Cl1—C12—C13—C14	176.93 (9)
C8—C3—C4—N1	179.41 (10)	C12—C13—C14—C15	0.59 (18)
C2—C3—C4—N1	−0.75 (12)	C13—C14—C15—C16	0.24 (19)
N1—C4—C5—C6	−179.77 (11)	C14—C15—C16—C11	0.07 (19)
C3—C4—C5—C6	0.78 (17)	C12—C11—C16—C15	−1.22 (17)
C4—C5—C6—F1	179.77 (11)	N1—C11—C16—C15	−179.44 (11)

### Hydrogen-bond geometry (Å, °)

**Table:**

Cg1 is the centroid of the C3–C8 benzene ring.

**Table:**

*D*—H···*A*	*D*—H	H···*A*	*D*···*A*	*D*—H···*A*
C7—H7···F1^i^	0.95	2.54	3.1638 (16)	123
C7—H7···Cl1^ii^	0.95	2.73	3.5296 (14)	142
C15—H15···Cg1^iii^	0.95	2.92	3.7246 (14)	143

Symmetry codes: (i) *x*+1/2, *y*, −*z*+3/2; (ii) −*x*+3/2, *y*+1/2, *z*; (iii) *x*−1, *y*, *z*.
